# Attention in Psychology, Neuroscience, and Machine Learning

**DOI:** 10.3389/fncom.2020.00029

**Published:** 2020-04-16

**Authors:** Grace W. Lindsay

**Affiliations:** Gatsby Computational Neuroscience Unit, Sainsbury Wellcome Centre, University College London, London, United Kingdom

**Keywords:** attention, artificial neural networks, machine learning, vision, memory, awareness

## Abstract

Attention is the important ability to flexibly control limited computational resources. It has been studied in conjunction with many other topics in neuroscience and psychology including awareness, vigilance, saliency, executive control, and learning. It has also recently been applied in several domains in machine learning. The relationship between the study of biological attention and its use as a tool to enhance artificial neural networks is not always clear. This review starts by providing an overview of how attention is conceptualized in the neuroscience and psychology literature. It then covers several use cases of attention in machine learning, indicating their biological counterparts where they exist. Finally, the ways in which artificial attention can be further inspired by biology for the production of complex and integrative systems is explored.

## 1. Introduction

Attention is a topic widely discussed publicly and widely studied scientifically. It has many definitions within and across multiple fields including psychology, neuroscience, and, most recently, machine learning (Chun et al., 2011; Cho et al., 2015). As William James wrote at the dawn of experimental psychology, “Everyone knows what attention is. It is the taking possession by the mind, in clear, and vivid form, of one out of what seems several simultaneously possible objects or trains of thought.” Since James wrote this, many attempts have been made to more precisely define and quantify this process while also identifying the underlying mental and neural architectures that give rise to it. The glut of different experimental approaches and conceptualizations to study what is spoken of as a single concept, however, has led to something of a backlash amongst researchers. As was claimed in the title of a recent article arguing for a more evolution-informed approach to the concept, “No one knows what attention is” (Hommel et al., 2019).

Attention is certainly far from a clear or unified concept. Yet despite its many, vague, and sometimes conflicting definitions, there is a core quality of attention that is demonstrably of high importance to information processing in the brain and, increasingly, artificial systems. Attention is the flexible control of limited computational resources. Why those resources are limited and how they can best be controlled will vary across use cases, but the ability to dynamically alter and route the flow of information has clear benefits for the adaptiveness of any system.

The realization that attention plays many roles in the brain makes its addition to artificial neural networks unsurprising. Artificial neural networks are parallel processing systems comprised of individual units designed to mimic the basic input-output function of neurons. These models are currently dominating the machine learning and artificial intelligence (AI) literature. Initially constructed without attention, various mechanisms for dynamically re-configuring the representations or structures of these networks have now been added.

The following section, section 2, will cover broadly the different uses of the word attention in neuroscience and psychology, along with its connection to other common neuroscientific topics. Throughout, the conceptualization of attention as a way to control limited resources will be highlighted. Behavioral studies will be used to demonstrate the abilities and limits of attention while neural mechanisms point to the physical means through which these behavioral effects are manifested. In section 3, the state of attention research in machine learning will be summarized and relationships between artificial and biological attention will be indicated where they exist. And in section 4 additional ways in which findings from biological attention can influence its artificial counterpart will be presented.

The primary aim of this review is to give researchers in the field of AI or machine learning an understanding of how attention is conceptualized and studied in neuroscience and psychology in order to facilitate further inspiration where fruitful. A secondary aim is to inform those who study biological attention how these processes are being operationalized in artificial systems as it may influence thinking about the functional implications of biological findings.

## 2. Attention in Neuroscience and Psychology

The scientific study of attention began in psychology, where careful behavioral experimentation can give rise to precise demonstrations of the tendencies and abilities of attention in different circumstances. Cognitive science and cognitive psychology aim to turn these observations into models of how mental processes could create such behavioral patterns. Many word models and computational models have been created that posit different underlying mechanisms (Driver, 2001; Borji and Itti, 2012).

The influence of single-cell neurophysiology in non-human primates along with non-invasive means of monitoring human brain activity such as EEG, fMRI, and MEG have made direct observation of the underlying neural processes possible. From this, computational models of neural circuits have been built that can replicate certain features of the neural responses that relate to attention (Shipp, 2004).

In the following sub-sections, the behavioral and neural findings of several different broad classes of attention will be discussed.

### 2.1. Attention as Arousal, Alertness, or Vigilance

In its most generic form, attention could be described as merely an overall level of alertness or ability to engage with surroundings. In this way it interacts with arousal and the sleep-wake spectrum. Vigilance in psychology refers to the ability to sustain attention and is therefore related as well. Note, while the use of these words clusters around the same meaning, they are sometimes used more specifically in different niche literature (Oken et al., 2006).

Studying subjects in different phases of the sleep-wake cycle, under sleep deprivation, or while on sedatives offers a view of how this form of attention can vary and what the behavioral consequences are. By giving subjects repetitive tasks that require a level of sustained attention—such as keeping a ball within a certain region on a screen—researchers have observed extended periods of poor performance in drowsy patients that correlate with changes in EEG signals (Makeig et al., 2000). Yet, there are ways in which tasks can be made more engaging that can lead to higher performance even in drowsy or sedated states. This includes increasing the promise of reward for performing the task, adding novelty or irregularity, or introducing stress (Oken et al., 2006). Therefore, general attention appears to have limited reserves that won't be deployed in the case of a mundane or insufficiently rewarding task but can be called upon for more promising or interesting work.

**Figure 1:**
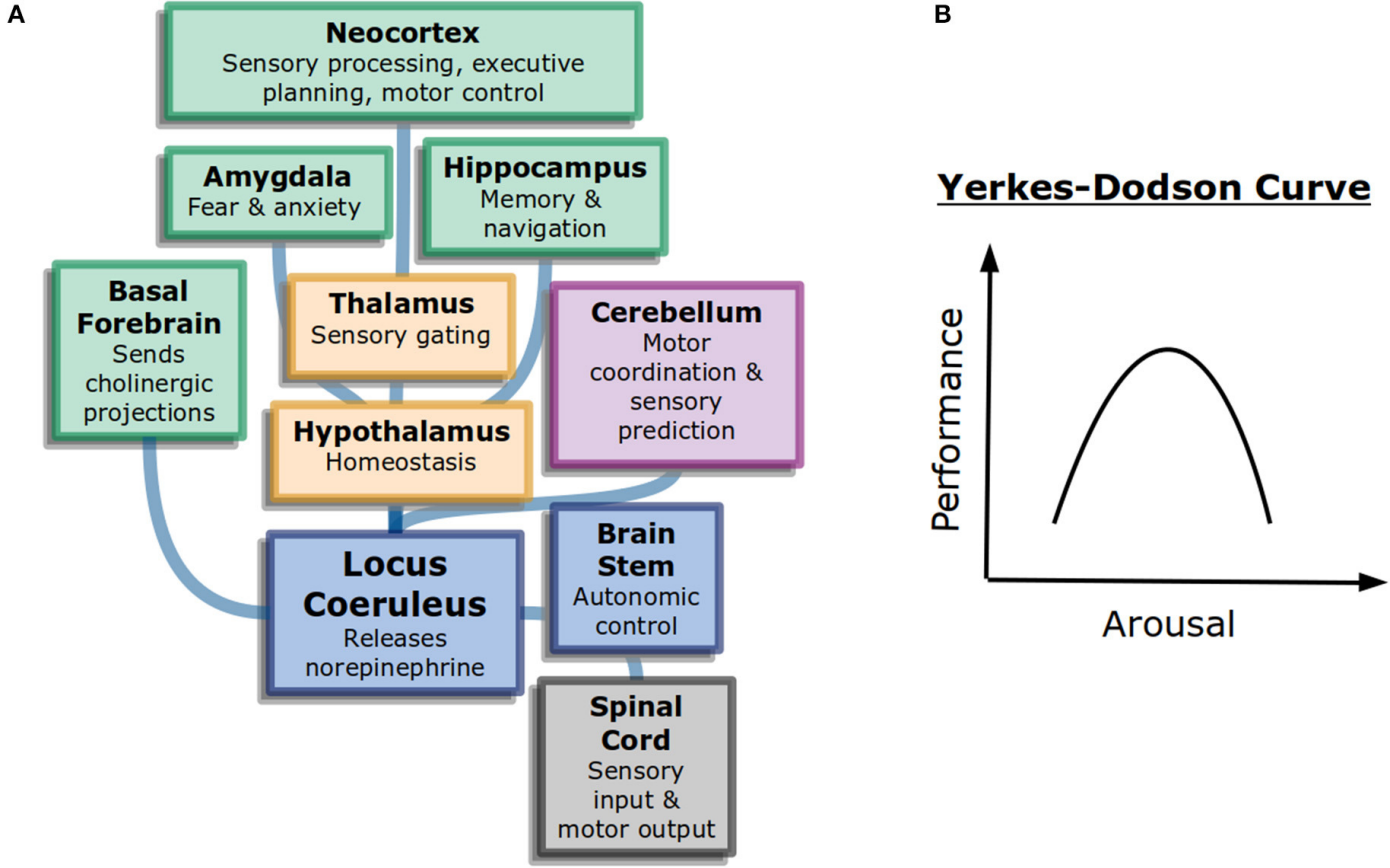
General attention and alertness **(A)** Cells in the locus coeruleus release norepinephrine (also known as noradrenaline) onto many parts of the brain with different functions, including onto other neuromodulatory systems. This contributes to overall arousal (Samuels and Szabadi, 2008). Colors here represent different divisions of the brain: forebrain (green), diencephalon (yellow), and brainstem (blue). **(B)** The Yerkes-Dodson curve describes the nonlinear relationship between arousal and performance on challenging tasks.

Interestingly, more arousal is not always beneficial. The Yerkes-Dodson curve (Figure 1B) is an inverted-U that represents performance as a function of alertness on sufficiently challenging tasks: at low levels of alertness performance is poor, at medium levels it is good, and at high levels it becomes poor again. The original study used electric shocks in mice to vary the level of alertness, but the finding has been repeated with other measures (Diamond, 2005). It may explain why psychostimulants such as Adderall or caffeine can work to increase focus in some people at some doses but become detrimental for others (Wood et al., 2014).

The neural circuits underlying the sleep-wake cycle are primarily in the brain stem (Coenen, 1998). These circuits control the flow of information into the thalamus and then onto cortex. Additionally, neuromodulatory systems play a large role in the control of generalized attention. Norepinephrine, acetylcholine, and dopamine are believed to influence alertnesss, orienting to important information, and executive control of attention, respectively (Posner, 2008). The anatomy of neuromodulators matches their function as well. Neurons that release norepinephrine, for example, have their cell bodies in the brain stem but project very broadly across the brain, allowing them to control information processing broadly (Figure 1A).

### 2.2. Sensory Attention

In addition to overall levels of arousal and alertness, attention can also be selectively deployed by an awake subject to specific sensory inputs. Studying attention within the context of a specific sensory system allows for tight control over both stimuli and the locus of attention. Generally, to look for this type of attention the task used needs to be quite challenging. For example, in a change detection task, the to-be-detected difference between two stimuli may be very slight. More generally, task difficulty can be achieved by presenting the stimulus for only a very short period of time or only very weakly.

A large portion of the study of attention in systems neuroscience and psychology centers on visual attention in particular (Kanwisher and Wojciulik, 2000). This may reflect the general trend in these fields to emphasis the study of visual processing over other sensory systems (Hutmacher, 2019), along with the dominant role vision plays in the primate brain. Furthermore, visual stimuli are frequently used in studies meant to address more general, cognitive aspects of attention as well.

Visual attention can be broken down broadly into spatial and feature-based attention.

#### 2.2.1. Visual Spatial Attention

Saccades are small and rapid eye movements made several times each second. As the fovea offers the highest visual resolution on the retina, choosing where to place it is essentially a choice about where to deploy limited computational resources. In this way, eye movements indicate the locus of attention. As this shift of attention is outwardly visible it is known as overt visual attention.

By tracking eye movements as subjects are presented with different images, researchers have identified image patterns that automatically attract attention. Such patterns are defined by oriented edges, spatial frequency, color contrast, intensity, or motion (Itti and Koch, 2001). Image regions that attract attention are considered “salient” and are computed in a “bottom-up” fashion. That is, they don't require conscious or effortful processing to identify and are likely the result of built-in feature detectors in the visual system. As such, saliency can be computed very quickly. Furthermore, different subjects tend to agree on which regions are salient, especially those identified in the first few saccades (Tatler et al., 2005).

Salient regions can be studied in “free-viewing” situations, that is, when the subject is not given any specific instructions about how to view the image. When a particular task is assigned, the interplay between bottom-up and “top-down” attention becomes clear. For example, when instructed to saccade to a specific visual target out of an array, subjects may incorrectly saccade to a particularly salient distractor instead (van Zoest and Donk, 2005). More generally, task instructions can have a significant effect on the pattern of saccades generated when subjects are viewing a complex natural image and given high-level tasks (e.g., asked to assess the age of a person or guess their socio-economic status). Furthermore, the natural pattern of eye movements when subjects perform real world tasks, like sandwich making, can provide insights to underlying cognitive processes (Hayhoe and Ballard, 2005).

When subjects need to make multiple saccades in a row they tend not to return to locations they have recently attended and may be slow to respond if something relevant occurs there. This phenomenon is known as inhibition of return (Itti and Koch, 2001). Such behavior pushes the visual system to not just exploit image regions originally deemed most salient but to explore other areas as well. It also means the saccade generating system needs to have a form of memory; this is believed to be implemented by short-term inhibition of the representation of recently-attended locations.

While eye movements are an effective means of controlling visual attention, they are not the only option. “Covert” spatial attention is a way of emphasizing processing of different spatial locations without an overt shift in fovea location. Generally, in the study of covert spatial attention, subjects must fixate on a central point throughout the task. They are cued to covertly attend to a location in their peripheral vision where stimuli relevant for their visual task will likely appear. For example, in an orientation discrimination task, after the spatial cue is provided an oriented grating will flash in the cued location and the subject will need to indicate its orientation. On invalidly-cued trials (when the stimulus appears in an uncued location), subjects perform worse than on validly-cued (or uncued) trials (Anton-Erxleben and Carrasco, 2013). This indicates that covert spatial attention is a limited resource that can be flexibly deployed and aids in the processing of visual information.

Covert spatial attention is selective in the sense that certain regions are selected for further processing at the expense of others. This has been referred to as the “spotlight” of attention. Importantly, for covert—as opposed to overt—attention the input to the visual system can be identical while the processing of that input is flexibly selective.

Covert spatial attention can be impacted by bottom-up saliency as well. If an irrelevant but salient object is flashed at a location that then goes on to have a task relevant stimulus, the exogenous spatial attention drawn by the irrelevant stimulus can get applied to the task relevant stimulus, possibly providing a performance benefit. If it is flashed at an irrelevant location, however, it will not help, and can harm performance (Berger et al., 2005). Bottom-up/exogenous attention has a quick time course, impacting covert attention for 80–130 ms after the distractor appears (Anton-Erxleben and Carrasco, 2013).

In some theories of attention, covert spatial attention exists to help guide overt attention. Particularly, the pre-motor theory of attention posits that the same neural circuits plan saccades and control covert spatial attention (Rizzolatti et al., 1987). The frontal eye field (FEF) is known to be involved in the control of eye movements. Stimulating the neurons in FEF at levels too low to evoke eye movements has been shown to create effects similar to covert attention (Moore et al., 2003). In this way, covert attention may be a means of deciding where to overtly look. The ability to covertly attend may additionally be helpful in social species, as eye movements convey information about knowledge and intent that may best be kept secret (Klein et al., 2009).

To study the neural correlates of covert spatial attention, researchers identify which aspects of neural activity differ based only on differences in the attentional cue (and not on differences in bottom-up features of the stimuli). On trials where attention is cued toward the receptive field of a recorded neuron, many changes in the neural activity have been observed (Noudoost et al., 2010; Maunsell, 2015). A commonly reported finding is an increase in firing rates, typically of 20–30% (Mitchell et al., 2007). However, the exact magnitude of the change depends on the cortical area studied, with later areas showing stronger changes (Luck et al., 1997; Noudoost et al., 2010). Attention is also known to impact the variability of neural firing. In particular, it decreases trial-to-trial variability as measured via the Fano Factor and decreases noise correlations between pairs of neurons. Attention has even been found to impact the electrophysiological properties of neurons in a way that reduces their likelihood of firing in bursts and also decreases the height of individual action potentials (Anderson et al., 2013).

In general, the changes associated with attention are believed to increase the signal-to-noise ratio of the neurons that represent the attended stimulus, however they can also impact communication between brain areas. To this end, attention's effect on neural synchrony is important. Within a visual area, attention has been shown to increase spiking coherence in the gamma band—that is at frequencies between 30 and 70 Hz (Fries et al., 2008). When a group of neurons fires synchronously, their ability to influence shared downstream areas is enhanced. Furthermore, attention may also be working to directly coordinate communication across areas. Synchronous activity between two visual areas can be a sign of increased communication and attention has been shown to increase synchrony between the neurons that represent the attended stimulus in areas V1 and V4, for example (Bosman et al., 2012). Control of this cross-area synchronization appears to be carried out by the pulvinar (Saalmann et al., 2012).

In addition to investigating how attention impacts neurons in the visual pathways, studies have also searched for the source of top-down attention (Noudoost et al., 2010; Miller and Buschman, 2014). The processing of bottom-up attention appears to culminate with a saliency map produced in the lateral intraparietal area (LIP). The cells here respond when salient stimuli are in their receptive field, including task-irrelevant but salient distractors. Prefrontal areas such as FEF, on the other hand, appear to house the signals needed for top-down control of spatial attention and are less responsive to distractors.

While much of the work on the neural correlates of sensory attention focuses on the cortex, subcortical areas appear to play a strong role in the control and performance benefits of attention as well. In particular, the superior colliculus assists in both covert and overt spatial attention and inactivation of this region can impair attention (Krauzlis et al., 2013). And, as mentioned above, the pulvinar plays a role in attention, particularly with respect to gating effects on cortex (Zhou et al., 2016).

#### 2.2.2. Visual Feature Attention

Feature attention is another form of covert selective attention. In the study of feature attention, instead of being cued to attend to a particular location, subjects are cued on each trial to attend to a particular visual feature such as a specific color, a particular shape, or a certain orientation. The goal of the task may be to detect if the cued feature is present on the screen or readout another one of its qualities (e.g., to answer “what color is the square?” should result in attention first deployed to squares). Valid cueing about the attended feature enhances performance. For example, when attention was directed toward a particular orientation, subjects were better able to detect faint gratings of that orientation than of any other orientation (Rossi and Paradiso, 1995). While the overall task (e.g., detection of an oriented grating) remains the same, the specific instructions (detection of 90° grating vs. 60° vs. 30°) will be cued on each individual trial, or possibly blockwise. Successful trial-wise cueing indicates that this form of attention can be flexibly deployed on fast timescales.

**Figure 2:**
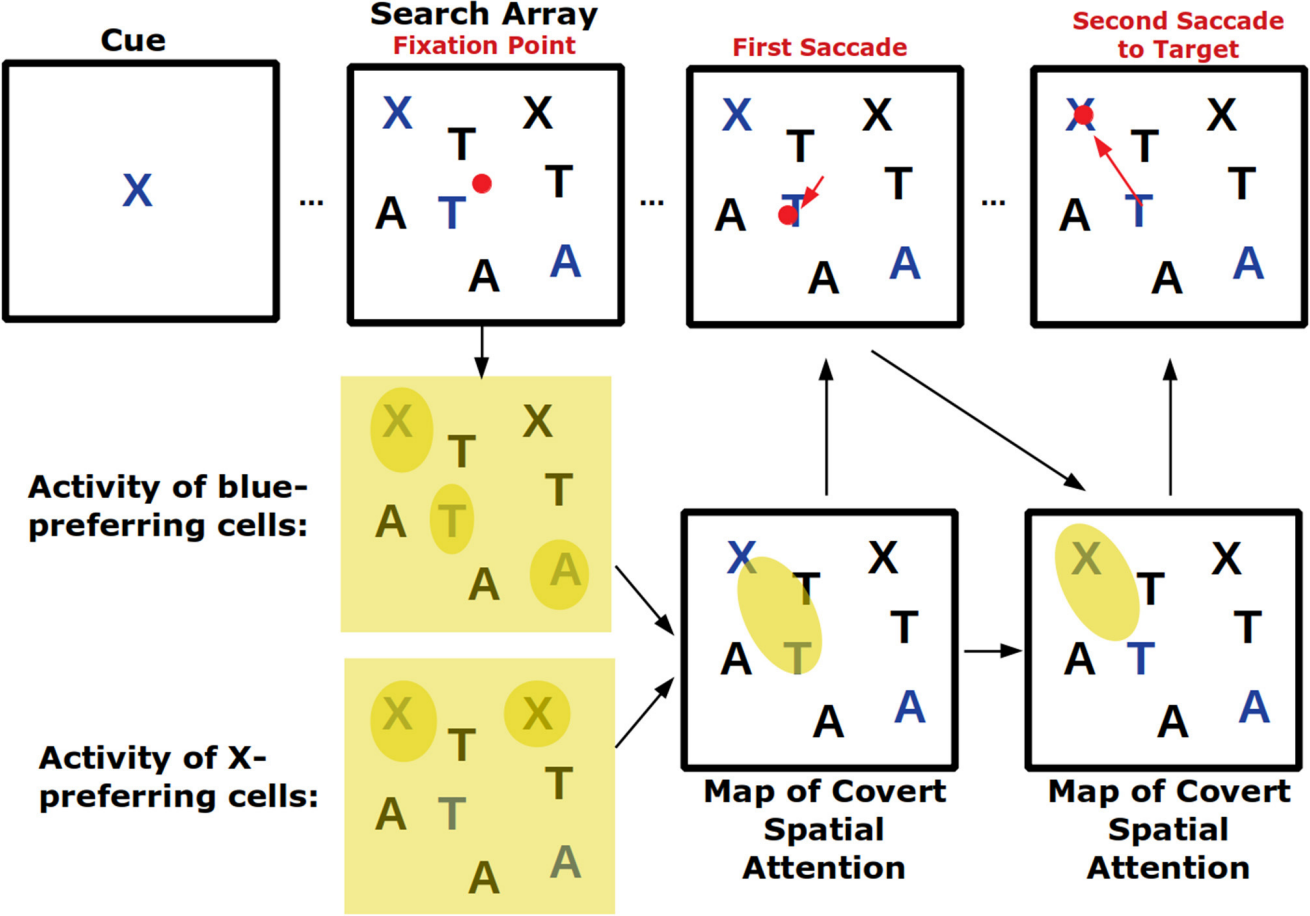
Visual search tasks engage many forms of visual attention. Across the top row the progression of a visual search task is shown. First, a cue indicates the target of the visual search, in this case a blue X. Then a search array appears with many non-targets. Top-down feature attention to cells that represent the color blue and the shape X will increase their firing throughout the visual field but firing will be strongest where blue or Xs actually occur. These neural response will play a role in generating a map of covert spatial attention which can be used to explore visual space before saccading. After the shift in overt attention with the first saccade, the covert attention map is remade. Finally, the target is located and successfully saccaded to. If the visual array contained a pop-out stimulus (for example a green O) it may have captured covert spatial attention in a bottom-up way and led to an additional incorrect saccade.

Visual search tasks are also believed to activate feature-based attention (Figure 2). In these tasks, an array of stimuli appears on a screen and subjects need to indicate—frequently with an eye movement—the location of the cued stimulus. As subjects are usually allowed to make saccades throughout the task as they search for the cued stimulus, this task combines covert feature-based attention with overt attention. In fact, signals of top-down feature-based attention have been found in FEF, the area involved in saccade choice (Zhou and Desimone, 2011). Because certain features can create a pop-out effect—for example, a single red shape amongst several black ones will immediately draw attention—visual search tasks also engage bottom-up attention which, depending on the task, may need to be suppressed (Wolfe and Horowitz, 2004).

Neural effects of feature-based attention in the visual system are generally similar to those of spatial attention. Neurons that represent the attended feature, for example, have increased firing rates, and those that represent very different features have suppressed rates (Treue and Trujillo, 1999). As opposed to spatial attention, however, feature-based attention is spatially-global. This means that when deploying attention to a particular feature the activity of the neurons that represent that feature anywhere in visual space are modulated (Saenz et al., 2002). Another difference between spatial and feature attention is the question of how sources of top-down attention target the correct neurons in the visual system. The retinotopic map, wherein nearby cells represent nearby spatial locations, makes spatial targeting straightforward, but cells are not as neatly organized according to preferred visual features.

The effects of spatial and feature attention appear to be additive (Hayden and Gallant, 2009). Furthermore, both feature and spatial attention are believed to create their effects by acting on the local neural circuits that implement divisive normalization in visual cortex (Reynolds and Heeger, 2009). Modeling work has shown that many of the neural effects of selective attention can be captured by assuming that top-down connections provide targeted synaptic inputs to cells in these circuits (Lindsay et al., 2019). However, models that rely on effects of the neuromodulator acetylcholine can also replicate neural correlates of attention (Sajedin et al., 2019).

Potential sources of top-down feature-based attention have been found in prefrontal cortex where sustained activity encodes the attended feature (Bichot et al., 2015; Paneri and Gregoriou, 2017). Inactivating the ventral prearcuate area impairs performance on search tasks. From prefrontal areas, attention signals are believed to travel in a reverse hierarchical way wherein higher visual areas send inputs to those below them (Ahissar and Hochstein, 2000).

A closely related topic to feature attention is object attention. Here, attention is not deployed to an abstract feature in advance of a visual stimulus, but rather it is applied to a particular object in the visual scene (Chen, 2012). The initial feedforward pass of activity through the visual hierarchy is able to pre-attentively segregate objects from their backgrounds in parallel across the visual field, provided these objects have stark and salient differences from the background. In more crowded or complex visual scenes, recurrent and serial processing is needed in order to identify different objects (Lamme and Roelfsema, 2000). Serial processing involves moving limited attentional resources from one location in the image to another; it can take the form of shifts in either covert or overt spatial attention (Buschman and Miller, 2009). Recurrent connections in the visual system—that is, both horizontal connections from nearby neurons in the same visual area and feedback connections from those in higher visual areas—aid in figure-ground segregation and object identification. The question of how the brain performs perceptual grouping of low-level features into a coherent object identity has been studied for nearly a century. It is believed that attention may be required for grouping, particularly for novel or complex objects (Roelfsema and Houtkamp, 2011). This may be especially important in visual search tasks that require locating an object that is defined by a conjunction of several features.

Neurally, the effects of object-based attention can spread slowly through space as parts of an object are mentally traced (Roelfsema et al., 1998). Switching attention to a location outside an object appears to incur a greater cost than switching to the same distance away but within the object (Brown and Denney, 2007). In addition, once attention is applied to a visual object, it is believed to activate feature-based attention for the different features of that object across the visual field (O'Craven et al., 1999).

Another form of attention sometimes referred to as feature attention involves attending to an entire feature dimension. An example of this is the Stroop test, wherein the names of colors are written in different colored ink and subjects either need to read the word itself or say the color of the ink. Here attention cannot be deployed to a specific feature in advance, only to the dimensions word or color. Neurally, the switch between dimensions appears to impact sensory coding in the visual stream and is controlled by frontal areas (Liu et al., 2003).

#### 2.2.3. Computational Models of Visual Attention

Visual attention, being one of the most heavily-studied topics in the neuroscience of attention, has inspired many computational models of how attention works. In general, these models synthesize various neurophysiological findings in order to help explain how the behavioral impacts of attention arise (Heinke and Humphreys, 2005).

Several computational models meant to calculate saliency have been devised (Itti and Koch, 2001). These models use low-level visual feature detectors—usually designed to match those in the visual system—to create an image-specific saliency map that can predict the saccade patterns of humans in response to the same image. Another approach to calculating saliency based on information theoretic first principles has also been explored and was able to account for certain visual search behaviors (Bruce and Tsotsos, 2009).

Some of the behavioral and neural correlates of attention are similar whether the attention is bottom-up or top-down. In the Biased Competition Model of attention, stimuli compete against each other to dominate the neural response (Desimone, 1998). Attention (bottom-up or top-down) can thus work by biasing this competition toward the stimulus that is the target of attention. While the Biased Competition Model is sometimes used simply as a “word model” to guide intuition, explicit computational instantiations of it have also been built. A hierarchical model of the visual pathway that included top-down biasing as well as local competition mediated through horizontal connections was able to replicate multiple neural effects of attention (Deco and Rolls, 2004). A model embodying similar principles but using spiking neurons was also implemented (Deco and Rolls, 2005).

Similar models have been constructed explicitly to deal with attribute naming tasks such as the Stroop test described above. The Selective Attention Model (SLAM), for example, has local competition in both the sensory encoding and motor output modules and can mimic known properties of response times in easier and more challenging Stroop-like tests (Phaf et al., 1990).

Visual perception has been framed and modeled as a problem of Bayesian inference (Lee and Mumford, 2003). Within this context, attention can help resolve uncertainty under settings where inference is more challenging, typically by modulating priors (Rao, 2005). For example, in Chikkerur et al. (2010) spatial attention functions to reduce uncertainty about object identity and feature attention reduces spatial uncertainty. These principles can capture both behavioral and neural features of attention and can be implemented in a biologically-inspired neural model.

The feature similarity gain model of attention (FSGM) is a description of the neural effects of top-down attention that can be applied in both the feature and spatial domain (Treue and Trujillo, 1999). It says that the way in which a neuron's response is modulated by attention depends on that neuron's tuning. Tuning is a description of how a neuron responds to different stimuli, so according to the FSGM a neuron that prefers (that is, responds strongly to), e.g., the color blue, will have its activity enhanced by top-down attention to blue. The FSGM also says attention to non-preferred stimuli will cause a decrease in firing and that, whether increased or decreased, activity is scaled multiplicatively by attention. Though not initially defined as a computational model, this form of neural modulation has since been shown through modeling to be effective at enhancing performance on challenging visual tasks (Lindsay and Miller, 2018).

Other models conceptualize attention as a dynamic routing of information through a network. An implementation of this form of attention can be found in the Selective Attention for Identification Model (SAIM) (Heinke and Humphreys, 2003). Here, attention routes information from the retina to a representation deemed the “focus of attention”; depending on the current task, different parts of the retinal representation will be mapped to the focus of attention.

#### 2.2.4. Attention in Other Sensory Modalities

A famous example of the need for selective attention in audition is the “cocktail party problem”: the difficulty of focusing on the speech from one speaker in a crowded room of multiple speakers and other noises (Bronkhorst, 2015). Solving the problem is believed to involve “early” selection wherein low level features of a voice such as pitch are used to determine which auditory information is passed on for further linguistic processing. Interestingly, selective auditory attention has the ability to control neural activity at even the earliest level of auditory processing, the cochlea (Fritz et al., 2007).

Spatial and feature attention have also been explored in the somatosensory system. Subjects cued to expect a tap at different parts on their body are better able to detect the sensation when that cue is valid. However, these effects seem weaker than they are in the visual system (Johansen-Berg and Lloyd, 2000). Reaction times are faster in a detection task when subjects are cued about the orientation of a stimulus on their finger (Schweisfurth et al., 2014).

In a study that tested subjects' ability to detect a taste they had been cued for it was shown that validly-cued tastes can be detected at lower concentrations than invalidly-cued ones (Marks and Wheeler, 1998). This mimics the behavioral effects found with feature-based visual attention. Attention to olfactory features has not been thoroughly explored, though visually-induced expectations about a scent can aid its detection (Gottfried and Dolan, 2003; Keller, 2011).

Attention can also be spread across modalities to perform tasks that require integration of multiple sensory signals. In general, the use of multiple congruent sensory signals aids detection of objects when compared to relying only on a single modality. Interestingly, some studies suggest that humans may have a bias for the visual domain, even when the signal from another domain is equally valid (Spence, 2009). Specifically, the visual domain appears to dominate most in tasks that require identifying the spatial location of a cue (Bertelson and Aschersleben, 1998). This can be seen most readily in ventriloquism, where the visual cue of the dummy's mouth moving overrides auditory evidence about the true location of the vocal source. Visual evidence can also override tactile evidence, for example, in the context of the rubber arm illusion (Botvinick and Cohen, 1998).

Another effect of the cross-modal nature of sensory processing is that an attentional cue in one modality can cause an orienting of attention in another modality (Spence and Driver, 2004). Generally, the attention effects in the non-cued modality are weaker. This cross-modal interaction can occur in the context of both endogenous (“top-down”) and exogenous (“bottom-up”) attention.

### 2.3. Attention and Executive Control

With multiple simultaneous competing tasks, a central controller is needed to decide which to engage in and when. What's more, how to best execute tasks can depend on history and context. Combining sensory inputs with past knowledge in order to coordinate multiple systems for the job of efficient task selection and execution is the role of executive control, and this control is usually associated with the prefrontal cortex (Miller and Buschman, 2014). As mentioned above, sources of top-down visual attention have also been located in prefrontal regions. Attention can reasonably be thought of as the output of executive control. The executive control system must thus select the targets of attention and communicate that to the systems responsible for implementing it. According to the reverse hierarchy theory described above, higher areas signal to those from which they get input which send the signal on to those below them and so on (Ahissar and Hochstein, 2000). This means that, at each point, the instructions for attention must be transformed into a representation that makes sense for the targeted region. Through this process, the high level goals of the executive control region can lead to very specific changes, for example, in early sensory processing.

Executive control and working memory are also intertwined, as the ability to make use of past information as well as to keep a current goal in mind requires working memory. Furthermore, working memory is frequently identified as sustained activity in prefrontal areas. A consequence of the three-way relationship between executive control, working memory, and attention is that the contents of working memory can impact attention, even when not desirable for the task (Soto et al., 2008). For example, if a subject has to keep an object in working memory while simultaneously performing a visual search for a separate object, the presence of the stored object in the search array can negatively interfere with the search (Soto et al., 2005). This suggests that working memory can interfere with the executive control of attention. However, there still appears to be additional elements of that control that working memory alone does not disrupt. This can be seen in studies wherein visual search performance is even worse when subjects believe they will need to report the memorized item but are shown a search array for the attended item instead (Olivers and Eimer, 2011). This suggests that, while all objects in working memory may have some influence over attention, the executive controller can choose which will have the most.

Beyond the flexible control of attention within a sensory modality, attention can also be shifted between modalities. Behavioral experiments indicate that switching attention either between two different tasks within a sensory modality (for example, going from locating a visual object to identifying it) or between sensory modalities (switching from an auditory task to a visual one) incurs a computational cost (Pashler, 2000). This cost is usually measured as the extent to which performance is worse on trials just after the task has been switched vs. those where the same task is being repeated. Interestingly, task switching within a modality seems to incur a larger cost than switching between modalities (Murray et al., 2009). A similar result is found when switching between or across modes of response (for example, pressing a bottom vs. verbal report), suggesting this is not specific to sensory processing (Arrington et al., 2003). Such findings are believed to stem from the fact that switching within a modality requires a reconfiguration of the same neural circuits, which is more difficult than merely engaging the circuitry of a different sensory system. An efficient executive controller would need to be aware of these costs when deciding to shift attention and ideally try to minimize them; it has been shown that switch costs can be reduced with training (Gopher, 1996).

The final question regarding the executive control of attention is how it evolves with learning. Eye movement studies indicate that searched-for items can be detected more rapidly in familiar settings rather than novel ones, suggesting that previously-learned associations guide overt attention (Chun and Jiang, 1998). Such benefits are believed to rely on the hippocampus (Aly and Turk-Browne, 2017). In general, however, learning how to direct attention is not as studied as other aspects of the attention process. Some studies have shown that subjects can enhance their ability to suppress irrelevant task information, and the generality of that suppression depends on the training procedure (Kelley and Yantis, 2009). Looking at the neural correlates of attention learning, imaging results suggest that the neural changes associated with learning do not occur in the sensory pathways themselves but rather in areas more associated with attentional control (Kelley and Yantis, 2010). Though not always easy to study, the development of attentional systems in infancy and childhood may provide further clues as to how attention can be learned (Reynolds and Romano, 2016).

### 2.4. Attention and Memory

Attention and memory have many possible forms of interaction. If memory has a limited capacity, for example, it makes sense for the brain to be selective about what is allowed to enter it. In this way, the ability of attention to dynamically select a subset of total information is well-matched to the needs of the memory system. In the other direction, deciding to recall a specific memory is a choice about how to deploy limited resources. Therefore, both memory encoding and retrieval can rely on attention.

The role of attention in memory encoding appears quite strong (Aly and Turk-Browne, 2017). For information to be properly encoded into memory, it is best for it be the target of attention. When subjects are asked to memorize a list of words while simultaneously engaging in a secondary task that divides their attention, their ability to consciously recall those words later is impaired (though their ability to recognize the words as familiar is not so affected) (Gardiner and Parkin, 1990). Imaging studies have shown that increasing the difficulty of the secondary task weakens the pattern of activity related to memory encoding in the left ventral inferior frontal gyrus and anterior hippocampus and increases the representation of secondary task information in dorsolateral prefrontal and superior parietal regions (Uncapher and Rugg, 2005). Therefore, without the limited neural processing power placed on the task of encoding, memory suffers. Attention has also been implicated in the encoding of spatially-defined memories and appears to stabilize the representations of place cells (Muzzio et al., 2009).

Implicit statistical learning can also be biased by attention. For example, in Turk-Browne et al. (2005) subjects watched a stream of stimuli comprised of red and green shapes. The task was to detect when a shape of the attended color appeared twice in a row. Unbeknownst to the subjects, certain statistical regularities existed in the stream such that there were triplets of shapes likely to occur close together. When shown two sets of three shapes—one an actual co-occurring triplet and another a random selection of shapes of the same color—subjects recognized the real triplet as more familiar, but only if the triplets were from the attended color. The statistical regularities of the unattended shapes were not learned.

Yet some learning can occur even without conscious attention. For example, in Watanabe (2003) patients engaged in a letter detection task located centrally in their visual field while random dot motion was shown in the background at sub-threshold contrast. The motion had 10% coherence in a direction that was correlated with the currently-presented letter. Before and after learning this task, subjects performed an above-threshold direction classification task. After learning the task, direction classification improved only for the direction associated with the targeted letters. This suggests a reward-related signal activated by the target led to learning about a non-attended component of the stimulus.

Many behavioral studies have explored the extent to which attention is needed for memory retrieval. For example, by asking subjects to simultaneously recall a list of previously-memorized words and engage in a secondary task like card sorting, researchers can determine if memory retrieval pulls from the same limited pool of attentional resources as the task. Some such studies have found that retrieval is impaired by the co-occurrence of an attention-demanding task, suggesting it is an attention-dependent process. The exact findings, however, depend on the details of the memory and non-memory tasks used (Lozito and Mulligan, 2006).

Even if memory retrieval does not pull from shared attentional resources, it is still clear that some memories are selected for more vivid retrieval at any given moment than others. Therefore, a selection process must occur. An examination of neuroimaging results suggests that the same parietal brain regions responsible for the top-down allocation and bottom-up capture of attention may play analogous roles during memory retrieval (Wagner et al., 2005; Ciaramelli et al., 2008).

Studies of memory retrieval usually look at medium to long-term memory but a mechanism for attention to items in working memory has also been proposed (Manohar et al., 2019). It relies on two different mechanisms of working memory: synaptic traces for non-attended items and sustained activity for the attended one.

Some forms of memory occur automatically and within the sensory processing stream itself. Priming is a well-known phenomenon in psychology wherein the presence of a stimulus at one point in time impacts how later stimuli are processed or interpreted. For example, the word “doctor” may be recognized more quickly following the word “hospital” than the word “school.” In this way, priming requires a form of implicit memory to allow previous stimuli to impact current ones. Several studies on conceptual or semantic priming indicate that attention to the first stimulus is required for priming effects to occur (Ballesteros and Mayas, 2015); this mirrors findings that attention is required for memory encoding more generally.

Most priming is positive, meaning that the presence of a stimulus at one time makes the detection and processing of it or a related stimulus more likely at a later time. In this way, priming can be thought of as biasing bottom-up attention. However, top-down attention can also create negative priming. In negative priming, when stimuli that functioned as a distractor on the previous trial serve as the target of attention on the current trial, performance suffers (Frings et al., 2015). This may stem from a holdover effect wherein the mechanisms of distractor suppression are still activated for the now-target stimulus.

Adaptation can also be considered a form of implicit memory. Here, neural responses decrease after repeated exposure to the same stimulus. By reducing the response to repetition, changes in the stimulus become more salient. Attention—by increasing the neural response to attended stimuli—counters the effects of adaptation (Pestilli et al., 2007; Anton-Erxleben et al., 2013). Thus, both with priming and adaptation, top-down attention can overcome automatic processes that occur at lower levels which may be guiding bottom-up attention.

## 3. Attention in Machine Learning

While the concept of artificial attention has come up prior to the current resurgence of artificial neural networks, many of its popular uses today center on ANNs (Mancas et al., 2016). The use of attention mechanisms in artificial neural networks came about—much like the apparent need for attention in the brain—as a means of making neural systems more flexible. Attention mechanisms in machine learning allow a single trained artificial neural network to perform well on multiple tasks or tasks with inputs of variable length, size, or structure. While the spirit of attention in machine learning is certainly inspired by psychology, its implementations do not always track with what is known about biological attention, as will be noted below.

In the form of attention originally developed for ANNs, attention mechanisms worked within an encoder-decoder framework and in the context of sequence models (Cho et al., 2015; Chaudhari et al., 2019). Specifically, an input sequence will be passed through an encoder (likely a recurrent neural network) and the job of the decoder (also likely a recurrent neural network) will be to output another sequence. Connecting the encoder and decoder is an attention mechanism.

Commonly, the output of the encoder is a set of a vectors, one for each element in the input sequence. Attention helps determine which of these vectors should be used to generate the output. Because the output sequence is dynamically generated one element at a time, attention can dynamically highlight different encoded vectors at each time point. This allows the decoder to flexibly utilize the most relevant parts of the input sequence.

The specific job of the attention mechanism is to produce a set of scalar weightings, $\alpha_{t}^{i}$, one for each of the encoded vectors (*v*^*i*^). At each step *t*, the attention mechanism (ϕ) will take in information about the decoder's previous hidden state (*h*_*t*−1_) and the encoded vectors to produce unnormalized weightings:

(1) $$\begin{array}{r} {\tilde{\alpha }}_{t}=\phi \left(h_{t-1},v\right) \end{array}$$

Because attention is a limited resource, these weightings need to represent relative importance. To ensure that the α values sum to one, the unnormalized weightings are passed through a softmax:

(2) $$\begin{array}{r} \alpha_{t}^{i}=\frac{\text{exp}\left({\tilde{\alpha }}_{t}^{i}\right)}{\sum_{j}\text{exp}\left({\tilde{\alpha }}_{t}^{j}\right)} \end{array}$$

These attention values scale the encoded vectors to create a single context vector on which the decoder can be conditioned:

(3) $$\begin{array}{r} c_{t}=\underset{j}{\sum }\alpha_{t}^{j}v^{j} \end{array}$$

This form of attention can be made entirely differentiable and so the whole network can be trained end-to-end with simple gradient descent.

This type of artificial attention is thus a form of iterative re-weighting. Specifically, it dynamically highlights different components of a pre-processed input as they are needed for output generation. This makes it flexible and context dependent, like biological attention. As such it is also inherently dynamic. While sequence modeling already has an implied temporal component, this form of attention can also be applied to static inputs and outputs (as will be discussed below in the context of image processing) and will thus introduce dynamics into the model.

In the traditional encoder-decoder framework without attention, the encoder produced a fixed-length vector that was independent of the length or features of the input and static during the course of decoding. This forced long sequences or sequences with complex structure to be represented with the same dimensionality as shorter or simpler ones and didn't allow the decoder to interrogate different parts of the input during the decoding process. But encoding the input as a set of vectors equal in length to the input sequence makes it possible for the decoder to selectively attend to the portion of the input sequence relevant at each time point of the decoding. Again, as in interpretations of attention in the brain, attention in artificial systems is helpful as a way to flexibly wield limited resources. The decoder can't reasonably be conditioned on the entirety of the input so at some point a bottleneck must be introduced. In the system without attention, the fixed-length encoding vector was a bottleneck. When an attention mechanism is added, the encoding can be larger because the bottleneck (in the form of the context vector) will be produced dynamically as the decoder determines which part of the input to attend to.

The motivation for adding such attention mechanisms to artificial systems is of course to improve their performance. But another claimed benefit of attention is interpretability. By identifying on which portions of the input attention is placed (that is, which α^*i*^ values are high) during the decoding process, it may be possible to gain an understanding of why the decoder produced the output that it did. However, caution should be applied when interpreting the outputs of attention as they may not always explain the behavior of the model as expected (Jain and Wallace, 2019; Wiegreffe and Pinter, 2019).

In the following subsections, specific applications of this general attention concept will be discussed, along with some that don't fit neatly into this framework. Further analogies to the biology will also be highlighted.

### 3.1. Attention for Natural Language Processing

As described above, attention mechanisms have frequently been added to models charged with processing sequences. Natural language processing (NLP) is one of the most common areas of application for sequence modeling. And, though it was not the original domain of attention in machine learning—nor does it have the most in common with biology—NLP is also one of the most common areas of application for attention (Galassi et al., 2019).

**Figure 3:**
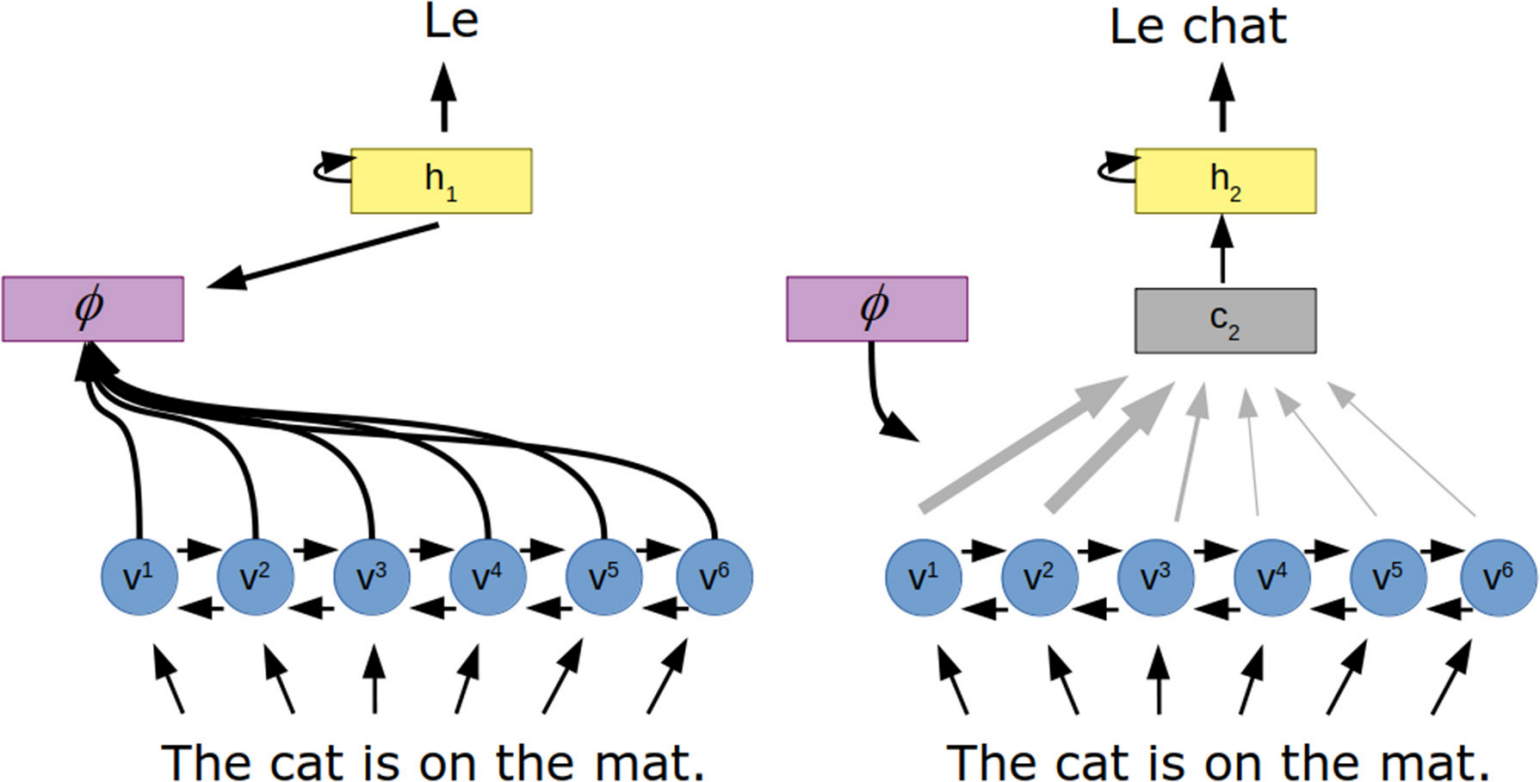
Attention for neural machine translation. The to-be-translated sentence is encoded to a series of vectors (*v*) via a recurrent neural network. The attention mechanism (ϕ) uses the hidden state of the decoder (*h*) and these vectors to determine how the encoded vectors should be combined to produce a context vector (*c*), which influences the next hidden state of the decoder and thus the next word in the translated sentence.

An early application of the this form of attention in artificial neural networks was to the task of translation (Bahdanau et al., 2014) (Figure 3). In this work, a recurrent neural network encodes the input sentence as a set of “annotation” vectors, one for each word in the sentence. The output, a sentence in the target language, is generated one word at a time by a recurrent neural network. The probability of each generated word is a function of the previously generated word, the hidden state of the recurrent neural network and a context vector generated by the attention mechanism. Here, the attention mechanism is a small feedforward neural network that takes in the hidden state of the output network as well as the current annotation vector to create the weighting over all annotation vectors.

Blending information from all the words in the sentence this way allows the network to pull from earlier or later parts when generating an output word. This can be especially useful for translating between languages with different standard word orders. By visualizing the locations in the input sentence to which attention was applied the authors observed attention helping with this problem.

Since this initial application, many variants of attention networks for language translation have been developed. In Firat et al. (2016), the attention mechanism was adapted so it could be used to translate between multiple pairs of languages rather than just one. In Luong et al. (2015), the authors explore different structures of attention to determine if the ability to access all input words at once is necessary. And in Cheng et al. (2016), attention mechanisms were added to the recurrent neural networks that perform the sentence encoding and decoding in order to more flexibly create sentence representations.

In 2017, the influential “Attention is All You Need” paper utilized a very different style of architecture for machine translation (Vaswani et al., 2017). This model doesn't have any recurrence, making it simpler to train. Instead, words in the sentence are encoded in parallel and these encodings generate key and query representations that are combined to create attention weightings. These weightings scale the word encodings themselves to create the next layer in the model, a process known as “self-attention.” This process repeats, and eventually interacts with the autoregressive decoder which also has attention mechanisms that allow it to flexibly focus on the encoded input (as in the standard form of attention) and on the previously generated output. The Transformer—the name given to this new attention architecture—outperformed many previous models and quickly became the standard for machine translation as well as other tasks (Devlin et al., 2018).

Interestingly, self-attention has less in common with biological attention than the recurrent attention models originally used for machine translation. First, it reduces the role of recurrence and dynamics, whereas the brain necessarily relies on recurrence in sequential processing tasks, including language processing and attentional selection. Second, self-attention provides a form of horizontal interaction between words—which allows for words in the encoded sentence to be processed in the context of those around them—but this mechanism does not include an obvious top-down component driven by the needs of the decoder. In fact, self-attention has been shown under certain circumstances to simply implement a convolution, a standard feedforward computation frequently used in image processing (Andreoli, 2019; Cordonnier et al., 2019). In this way, self-attention is more about creating a good encoding than performing a task-specific attention-like selection based on limited resources. In the context of a temporal task, its closest analogue in psychology may be priming because priming alters the encoding of subsequent stimuli based on those that came before. It is of course not the direct goal of machine learning engineers to replicate the brain, but rather to create networks that can be easily trained to perform well on tasks. These different constraints mean that even large advances in machine learning do not necessarily create more brain-like models.

While the study of attention in human language processing is not as large as other areas of neuroscience research, some work has been done to track eye movements while reading (Myachykov and Posner, 2005). They find that people will look back at previous sections of text in order to clarify what they are currently reading, particularly in the context of finding the antecedent of a pronoun. Such shifts in overt attention indicate what previous information is most relevant for the current processing demands.

### 3.2. Attention for Visual Tasks

As in neuroscience and psychology, a large portion of studies in machine learning are done on visual tasks. One of the original attention-inspired tools of computer vision is the saliency map, which identifies which regions in an image are most salient based on a set of low-level visual features such as edges, color, or depth and how they differ from their surround (Itti and Koch, 2001). In this way, saliency maps indicate which regions would be captured by “bottom-up” attention in humans and animals. Computer scientists have used saliency maps as part of their image processing pipeline to identify regions for further processing.

In more recent years, computer vision models have been dominated by deep learning. And since their success in the 2012 ImageNet Challenge (Russakovsky et al., 2015), convolutional neural networks have become the default architecture for visual tasks in machine learning.

The architecture of convolutional neural networks is loosely based on the mammalian visual system (Lindsay, 2020). At each layer, a bank of filters is applied to the activity of the layer below (in the first layer this is the image). This creates a *H* × *W* × *C* tensor of neural activity with the number of channels, *C* equal to the number of filters applied and *H* and *W* representing the height and width of the 2-D feature maps that result from the application of a filter.

Attention in convolutional neural networks has been used to enhance performance on a variety of tasks including classification, segmentation, and image-inspired natural language processing. Also, as in the neuroscience literature, these attentional processes can be divided into spatial and feature-based attention.

#### 3.2.1. Spatial Attention

Building off of the structures used for attention in NLP tasks, visual attention has been applied to image captioning. In Xu et al. (2015), the encoding model is a convolutional neural network. The attention mechanism works over the activity at the fourth convolutional layer. As each word of the caption is generated, a different pattern of weighting across spatial locations of the image representation is created. In this way, attention for caption generation replaces the set of encoded word vectors in a translation task with a set of encoded image locations. Visualizing the locations with high weights, the model appears to attend to the object most relevant to the current word being generated for the caption.

**Figure 4:**
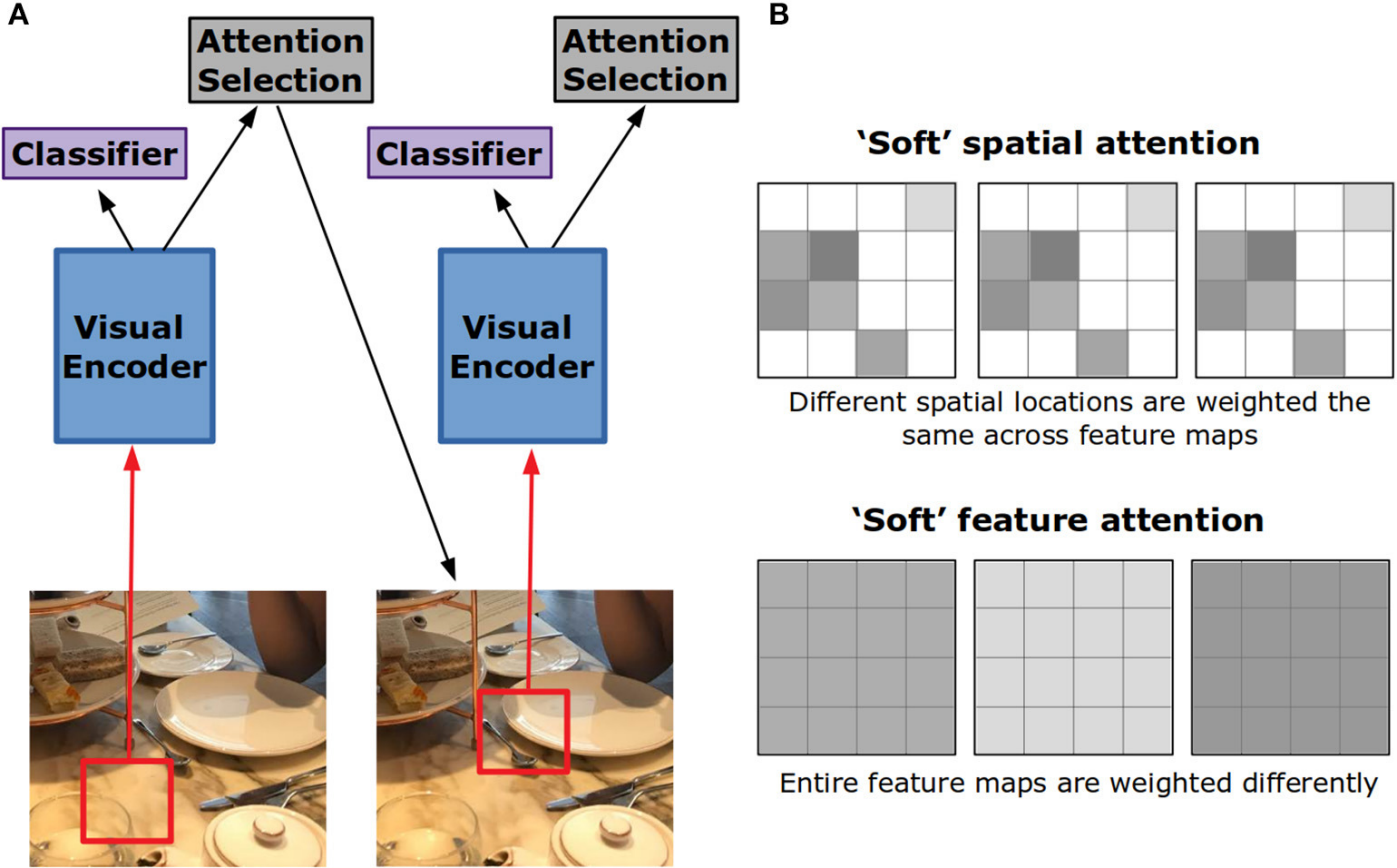
Hard vs. soft visual attention in artificial neural networks. **(A)** In hard attention, the network only gets input from a small portion of the whole image. This portion is iteratively chosen by the network through an attention selection mechanism. If the input is foveated, the network can use the lower resolution periphery to guide this selection. **(B)** Feature maps in convolutional neural networks are 2-D grids of activation created by the application of a filter to the layer below. In soft spatial attention, different locations on these grids are weighted differently. In soft feature attention, different feature maps are weighted differently.

This style of attention is referred to as “soft” because it produces a weighted combination of the visual features over spatial locations (Figure 4B). “Hard” attention is an alternative form that chooses a single spatial location to be passed into the decoder at the expense of all others (Figure 4A). In Xu et al. (2015), to decide which location should receive this hard attention, the attention weights generated for each spatial location were treated as probabilities. One location is chosen according to these probabilities. Adding this stochastic element to the network makes training more difficult, yet it was found to perform somewhat better than soft attention.

A 2014 study used reinforcement learning to train a hard attention network to perform object recognition in challenging conditions (Mnih et al., 2014). The core of this model is a recurrent neural network that both keeps track of information taken in over multiple “glimpses” made by the network and outputs the location of the next glimpse. For each glimpse, the network receives a fovea-like input (central areas are represented with high resolution and peripheral with lower) from a small patch of the image. The network has to integrate the information gained from these glimpses to find and classify the object in the image. This is similar to the hard attention described above, except the selection of a location here determines which part of the image is sampled next (whereas in the case above it determined which of the already-processed image locations would be passed to the decoder). With the use of these glimpses, the network is not required to process all of the image, saving computational resources. It can also help when multiple objects are present in the image and the network must classify each (Ba et al., 2014). Recent work has shown that adding a pre-training step enhances the performance of hard attention applied to complex images (Elsayed et al., 2019).

In many ways, the correspondence between biological and artificial attention is strongest when it comes to visual spatial attention. For example, this form of hard attention—where different locations of the image are sequentially-sampled for further processing—replicates the process of saccading and is therefore akin to overt visual attention in the neuroscience and psychology literature. Insofar as soft attention dynamically re-weights different regions of the network's representation of the image without any change in the input to the network, it is akin to covert spatial attention. Also, as the mode of application for soft attention involves multiplicative scaling of the activity of all units at a specific location, it replicates neural findings about covert spatial attention.

Soft spatial attention has been used for other tasks, including visual question and answering (Chen et al., 2015; Xu and Saenko, 2016; Yang et al., 2016) and action recognition in videos (Sharma et al., 2015). Hard attention has also been used for instance segmentation (Ren and Zemel, 2017) and for fine-grained classification when applied using different levels of image resolution (Fu et al., 2017).

#### 3.2.2. Feature Attention

In the case of soft spatial attention, weights are different in different spatial locations of the image representation yet they are the same across all feature channels at that location. That is, the activity of units in the network representing different visual features will all be modified the same way if they represent the same location in image space. Feature attention makes it possible to dynamically re-weight individual feature maps, creating a spatially global change in feature processing.

In Stollenga et al. (2014), a convolutional neural network is equipped with a feature-based attention mechanism. After an image is passed through the standard feedforward architecture, the activity of the network is passed into a policy that determines how the different feature maps at different layers should be weighted. This re-weighting leads to different network activity which leads to different re-weightings. After the network has run for several timesteps the activity at the final layer is used to classify the object in the image. The policy that determines the weighting values is learned through reinforcement learning, and can be added to any pre-trained convolutional neural network.

The model in Chen et al. (2017) combines feature and spatial attention to aid in image captioning. The activity of the feedforward pass of the convolutional network is passed into the attention mechanism along with the previously generated word to create attention weightings for different channels at each layer in the CNN. These weights are used to scale activity and then a separate attention mechanism does the same procedure for generating spatial weightings. Both spatial and feature attention weights are generated and applied to the network at each time point.

In the model in De Vries et al. (2017), the content of a question is used to control how a CNN processes an image for the task of visual question and answering. Specifically, the activity of a language embedding network is passed through a multi-layer perceptron to produce the additive and multiplicative parameters for batch normalization of each channel in the CNN. This procedure, termed conditional batch normalization, functions as a form of question-dependent feature attention.

A different form of dynamic feature re-weighting appears in “squeeze-and-excitation” networks (Hu et al., 2018). In this architecture, the weightings applied to different channels are a nonlinear function of the activity of the other channels at the same layer. As with “self-attention” described above, this differs in spirit from more “top-down” approaches where weightings are a function of activity later in the network and/or biased by the needs of the output generator. Biologically speaking, this form of interaction is most similar to horizontal connections within a visual area, which are known to carry out computations such as divisive normalization (Carandini and Heeger, 2012).

In the study of the biology of feature-based attention, subjects are usually cued to attend to or search for specific visual features. In this way, the to-be-attended features are known in advance and relate to the specific sub-task at hand (e.g., detection of a specific shape on a given trial of a general shape detection task). This differs from the above instances of artificial feature attention, wherein no external cue biases the network processing before knowledge about the specific image is available. Rather, the feature re-weighting is a function of the image itself and meant to enhance the performance of the network on a constant task (note this was also the case for the forms of artificial spatial attention described).

The reason for using a cueing paradigm in studies of biological attention is that it allows the experimenter to control (and thus know) where attention is placed. Yet, it is clear that even without explicit cueing, our brains make decisions about where to place attention constantly; these are likely mediated by local and long-range feedback connections to the visual system (Wyatte et al., 2014). Therefore, while the task structure differs between the study of biological feature attention and its use in artificial systems, this difference may only be superficial. Essentially, the artificial systems are using feedforward image information to internally generate top-down attentional signals rather than being given the top-down information in the form of a cue.

That being said, some artificial systems do allow for externally-cued feature attention. For example setting a prior over categories in the network in Cao et al. (2015) makes it better at localizing the specific category. The network in Wang et al. (2014), though not convolutional, has a means of biasing the detection of specific object categories as well. And in Lindsay and Miller (2018), several performance and neural aspects of biological feature attention during a cued object detection task were replicated using a CNN. In Luo et al. (2020), the costs and benefits of using a form of cued attention in CNNs were explored.

As mentioned above, the use of multiplicative scaling of activity is in line with certain findings from biological visual attention. Furthermore, modulating entire feature maps by the same scalar value is aligned with the finding mentioned above that feature attention acts in a spatially global way in the visual system.

### 3.3. Multi-Task Attention

Multi-task learning is a challenging topic in machine learning. When one network is asked to perform several different tasks—for example, a CNN that must classify objects, detect edges, and identify salient regions—training can be difficult as the weights needed to do each individual task may contradict each other. One option is have a set of task-specific parameters that modulate the activity of the shared network differently for each task. While not always called it, this can reasonably be considered a form of attention, as it flexibly alters the functioning of the network.

In Maninis et al. (2019), a shared feedforward network is trained on all of multiple tasks, while task specific skip connections and squeeze-and-excitation blocks are trained to modulate this activity only on their specific task. This lets the network benefit from sharing processing that is common to all tasks while still specializing somewhat to each.

A similar procedure was used in Rebuffi et al. (2017) to create a network that performs classification on multiple different image domains. There, the domain could be identified from the input image making it possible to select the set of task-specific parameters automatically at run-time.

In Zhao et al. (2018), the same image can be passed into the network and be classified along different dimensions (e.g. whether the person in the picture is smiling or not, young or old). Task-specific re-weighting of feature channels is used to execute these different classifications.

The model in Strezoski et al. (2019) uses what could be interpreted as a form of hard feature attention to route information differently in different tasks. Binary masks over feature channels are chosen randomly for each task. These masks are applied in a task-specific way during training on all tasks and at run-time. Note that in this network no task-specific attentional parameters are learned, as these masks are pre-determined and fixed during training. Instead, the network learns to use the different resulting information pathways to perform different tasks.

In a recent work, the notion of task-specific parameters was done away with entirely (Levi and Ullman, 2020). Instead, the activations of a feedforward CNN are combined with a task input and passed through a second CNN to generate a full set of modulatory weights. These weights then scale the activity of the original network in a unit-specific way (thus implementing both spatial and feature attention). The result is a single set of feedforward weights capable of flexibly engaging in multiple visual tasks.

When the same input is processed differently according to many different tasks, these networks are essentially implementing a form of within-modality task switching that relies on feature attention. In this way, it is perhaps most similar to the Stroop test described previously.

### 3.4. Attention to Memory

Deep neural networks tend not to have explicit memory, and therefore attention to memory is not studied. Neural Turing Machines, however, are a hybrid neural architecture that includes external memory stores (Graves et al., 2014). The network, through training, learns how to effectively interact with these stores to perform tasks such as sorting and repetition of stored sequences. Facilitating this interaction is a form of attention. Memories are stored as a set of vectors. To retrieve information from this store, the network generates a weight for each vector and calculates a weighted sum of the memories. To determine these weights, a recurrent neural network (which receives external and task-relevant input) outputs a vector and memories are weighted in accordance to their similarity to this vector. Thus, at each point in time, the network is able to access context-relevant memories.

As described previously, how the brain chooses what memories to attend to and then attends to them is not entirely clear. The use of a similarity metric in this model means that memories are retrieved based on their overlap with a produced activity vector, similar to associative memory models in the neuroscience literature. This offers a mechanism for the latter question—that is, how attention to memory could be implemented in the brain. The activity vector that the model produces controls what memories get attended and the relationship with biology is less clear here.

## 4. Ideas for Future Interaction Between Artificial and Biological Attention

**Figure 5:**
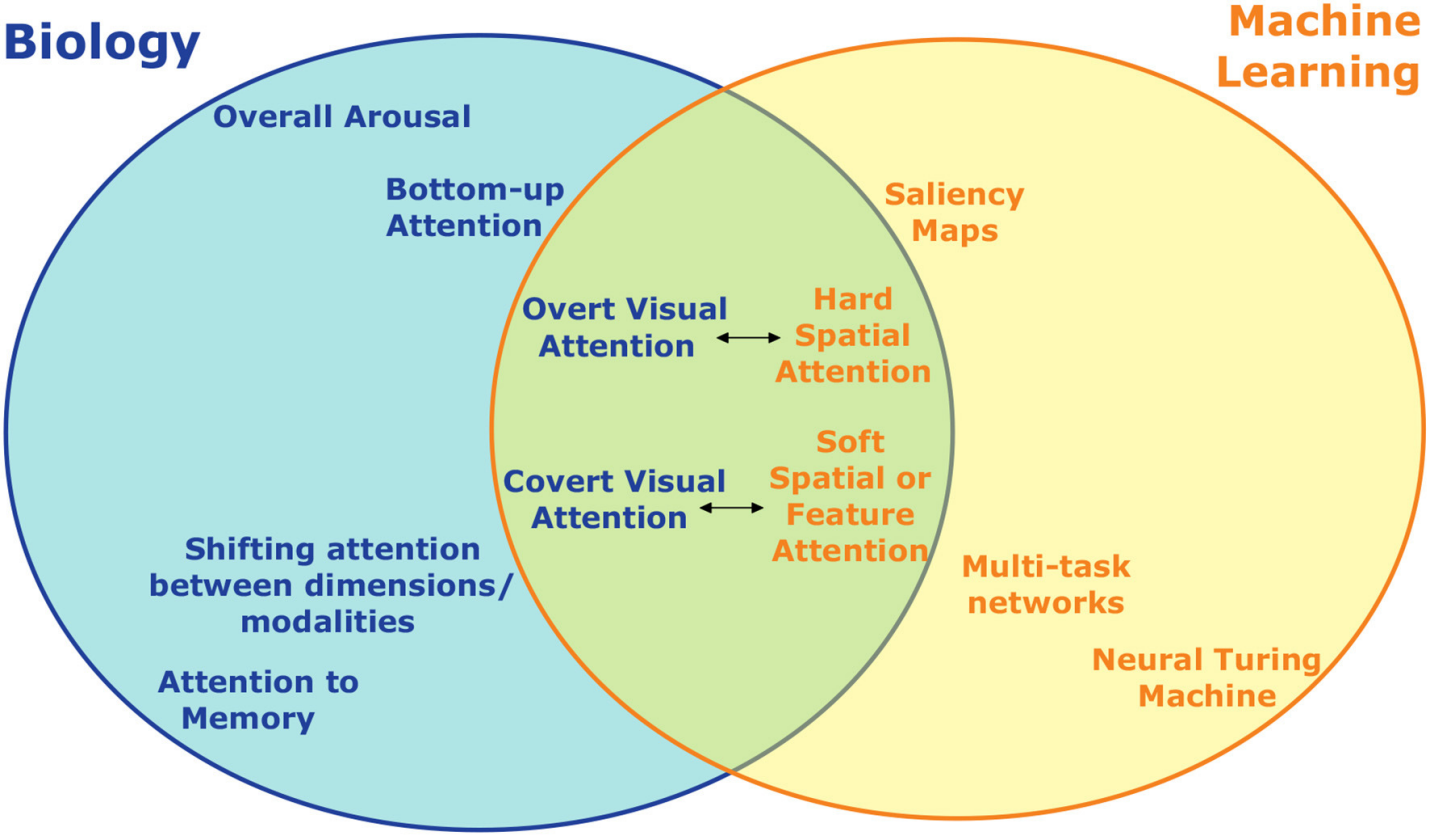
An incomplete summary of the different types of attention studied in neuroscience/psychology and machine learning and how they relate. On the left are divisions of attention studied biologically, on the right are those developed for artificial intelligence and machine learning. Topics at the same horizontal location are to some extent analogous, with the distance between them indicating how close the analogy is. Forms of visual attention, for example, have the most overlap and are the most directly comparable across biology and machine learning. Some forms of attention, such as overall arousal, don't have an obvious artificial analogue.

As has been shown, some amount of inspiration from biology has already led to several instances of attention in artificial neural networks (summarized in Figure 5). While the addition of such attention mechanisms has led to appreciable increases in performance in these systems, there are clearly still many ways in which they fall short and additional opportunities for further inspiration exist. In the near term, this inspiration will likely be in the form of incremental improvements to specialized artificial systems as exist now. However, the true promise of brain-inspired AI should deliver a more integrated, multiple-purpose agent that can engage flexibly in many tasks.

### 4.1. How to Enhance Performance

There are two components to the study of how attention works in the brain that can be considered flip sides of the same coin. The first is the question of how attention enhances performance in the way that it does—that is, how do the neural changes associated with attention make the brain better at performing tasks. The second is how and why attention is deployed in the way that it is—what factors lead to the selection of certain items or tasks for attention and not others.

Neuroscientists have spent a lot of time investigating the former question. In large part, the applicability of these findings to artificial neural systems, however, may not be straightforward. Multiplicative scaling of activity appears in both biological and artificial systems and is an effective means of implementing attention. However, many of the observed effects of attention in the brain make sense mainly as a means of increasing the signal carried by noisy, spiking neurons. This includes increased synchronization across neurons and decreased firing variability. Without analogs for these changes in deep neural networks, it is hard to take inspiration from them. What's more, the training procedures for neural networks can automatically determine the changes in activity needed to enhance performance on a well-defined task and so lessons from biological changes may not be as relevant.

On the other hand, the observation that attention can impact spiking-specific features such as action potential height, burstiness, and precise spike times may indicate the usefulness of spiking networks. Specifically, spiking models offer more degrees of freedom for attention to control and thus allow attention to possibly have larger and/or more nuanced impacts.

Looking at the anatomy of attention may provide usable insights to people designing architectures for artificial systems. For example, visual attention appears to modulate activity more strongly in later visual areas like V4 (Noudoost et al., 2010), whereas auditory attention can modulate activity much earlier in the processing stream. The level at which attention should act could thus be a relevant architectural variable. In this vein, recent work has shown that removing self-attention from the early layers of a Transformer model enhances its performance on certain natural language processing tasks and also makes the model a better predictor of human fMRI signals during language processing (Toneva and Wehbe, 2019).

The existence of cross-modal cueing—wherein attention cued in one sensory modality can cause attention to be deployed to the same object or location in another modality—indicates some amount of direct interaction between different sensory systems. Whereas many multi-modal models in machine learning use entirely separate processing streams that are only combined at the end, allowing some horizontal connections between different input streams may help coordinate their processing.

Attention also interacts with the kind of adaptation that normally occurs in sensory processing. Generally, neural network models do not have mechanisms for adaptation—that is, neurons have no means of reducing their activity if given the same input for multiple time steps. Given that adaptation helps make changes and anomalies stand out, it may be useful to include. In a model with adaption, attention mechanisms should work to reactivate adapted neurons if the repeated stimulus is deemed important.

Finally, some forms of attention appear to act in multiple ways on the same system. For example, visual attention is believed to both: (1) enhance the sensitivity of visual neurons in the cortex by modulating their activity and (2) change subcortical activity such that sensory information is readout differently (Birman and Gardner, 2019; Sreenivasan and Sridharan, 2019). In this way, attention uses two different mechanisms, in different parts of the brain, to create its effect. Allowing attention to modulate multiple components of a model architecture in complementary ways may allow it to have more robust and effective impacts.

### 4.2. How to Deploy Attention

The question of how to deploy attention is likely the more relevant challenge for producing complex and integrated artificial intelligence. Choosing the relevant information in a stream of incoming stimuli, picking the best task to engage in, or deciding whether to engage in anything at all requires that an agent have an integrative understanding of its state, environment, and needs.

The most direct way to take influence from biological attention is to mimic it directly. Scanpath models, for example, have existed in the study of saliency for many years. They attempt to predict the series of fixations that humans make while viewing images (Borji and Itti, 2019). A more direct approach to training attention was used in Linsley et al. (2018). Here, a large dataset of human top-down attention was collected by having subjects label the regions of images most relevant for object classification. The task-specific saliency maps created through this method were used to train attention in a deep convolutional neural network whose main task was object recognition. They found that influencing the activity of intermediate layers with this method could increase performance. Another way of learning a teacher's saliency map was given in Zagoruyko and Komodakis (2016).

Combined training on tasks and neural data collected from human visual areas has also helped the performance of CNNs (Fong et al., 2018). Using neural data collected during attention tasks in particular could help train attention models. Such transfer could also be done for other tasks. For example, tracking eye movements during reading could inform NLP models; thus far, eye movements have been used to help train a part-of-speech tagging model (Barrett et al., 2016). Interestingly, infants may learn from attending to what adults around them attend to and the coordination of attention more broadly across agents may be very helpful in a social species. Therefore, the attention of others should influence how attention is guided. Attempts to coordinate joint attention will need to be integrated into attention systems (Kaplan and Hafner, 2006; Klein et al., 2009).

Activities would likely need to flexibly decide which of several possible goals should be achieved at any time and therefore where attention should be placed. This problem clearly interacts closely with issues around reinforcement learning—particularly hierarchical reinforcement learning which involves the choosing of subtasks—as such decisions must be based on expected positive or negative outcomes. Indeed, there is a close relationship between attention and reward as previously rewarded stimuli attract attention even in contexts where they no longer provide reward (Camara et al., 2013). A better understanding of how humans choose which tasks to engage in and when should allow human behavior to inform the design of a multi-task AI.

To this end, the theory put forth in Shenhav et al. (2013), which says that allocation of the brain's limited ability to control different processes is based on the expected value of that control, may be of use. In this framework, the dorsal anterior cingulate cortex is responsible for integrating diverse information—including the cognitive costs of control—in order to calculate the expected value of control and thus direct processes like attention. Another approach for understanding human executive control in complex tasks is inverse reinforcement learning. This method was recently applied to a dataset of eye movements during visual search in order to determine the reward functions and policies used by humans (Zelinsky et al., 2020).

An additional factor that drives biological attention but is perhaps underrepresented in artificial attention systems is curiosity (Gottlieb et al., 2013). In biology, novel, confusing, and surprising stimuli can grab attention, and inferotemporal and perirhinal cortex are believed to signal novel visual situations via an adaptation mechanism that reduces responses to familiar inputs. Reinforcement learning algorithms that include novelty as part of the estimate of the value of a state can encourage this kind of exploration (Jaegle et al., 2019). How exactly to calculate surprise or novelty in different circumstances is not always clear, however. Previous work on biological attention has understood attention selection in Bayesian terms of surprise or information gathering and these framings may be useful for artificial systems (Itti and Baldi, 2006; Mirza et al., 2019).

A final issue in the selection of attention is how conflicts are resolved. Given the brain's multiple forms of attention—arousal, bottom-up, top-down, etc.—how do conflicts regarding the appropriate locus of attention get settled? Looking at the visual system, it seems that the local circuits that these multiple systems target are burdened with this task. These circuits receive neuromodulatory input along with top-down signals which they must integrate with the bottom-up input driving their activity. Horizontal connections mediate this competition, potentially using winner-take-all mechanisms. This can be mimicked in the architecture of artificial systems.

### 4.3. Attention and Learning

Attention, through its role in determining what enters memory, guides learning. Most artificial systems with attention include the attention mechanism throughout training. In this way, the attention mechanism is trained along with the base architecture; however, with the exception of the Neural Turing Machine, the model does not continue learning once the functioning attention system is in place. Therefore, the ability of attention to control learning and memory is still not explicitly considered in these systems.

Attention could help make efficient use of data by directing learning to the relevant components and relationships in the input. For example, saliency maps have been used as part of the pre-processing for various computer vision tasks (Lee et al., 2004; Wolf et al., 2007; Bai and Wang, 2014). Focusing subsequent processing only on regions that are intrinsically salient can prevent wasteful processing on irrelevant regions and, in the context of network training, could also prevent overfitting to these regions. Using saliency maps in this way, however, requires a definition of saliency that works for the problem at hand. Using the features of images that capture bottom-up attention in humans has worked for some computer vision problems; looking at human data in other modalities may be useful as well.

In a related vein, studies on infants suggest that they have priors that guide their attention to relevant stimuli such as faces. Using such priors could bootstrap learning both of how to process important stimuli and how to better attend to their relevant features (Johnson, 2001).

In addition to deciding which portions of the data to process, top-down attention can also be thought of as selecting which elements of the network should be most engaged during processing. Insofar as learning will occur most strongly in the parts of the network that are most engaged, this is another means by which attention guides learning. Constraining the number of parameters that will be updated in response to any given input is an effective form of regularization, as can be seen in the use of dropout and batch normalization. Attention—rather than randomly choosing which units to engage and disengage—is constrained to choose units that will also help performance on this task. It is therefore a more task-specific form of regularization.

In this way, attention may be particularly helpful for continual learning where the aim is to update a network to perform better on a specific task while not disrupting performance on the other tasks the network has already learned to do. A related concept, conditional computation, has recently been applied to the problem of continual learning (Lin et al., 2019). In conditional computation, the parameters of a network are a function of the current input (it can thus be thought of as an extreme form of the type of modulation done by attention); optimizing the network for efficient continual learning involves controlling the amount of interference between different inputs. More generically, it may be helpful to think of attention, in part, as a means of guarding against undesirable synaptic changes.

Attention and learning also work in a loop. Specifically, attention guides what is learned about the world and internal world models are used to guide attention. This inter-dependency has recently been formalized in terms of a reinforcement learning framework that also incorporates cognitive Bayesian inference models that have succeeded in explaining human learning and decision making (Radulescu et al., 2019). Interconnections between basal ganglia and prefrontal cortex are believed to support the interplay between reinforcement learning and attention selection.

At a more abstract level, the mere presence of attention in the brain's architecture can influence representation learning. The global workspace theory of consciousness says that at any moment a limited amount of information selected from the brain's activity can enter working memory and be available for further joint processing (Baars, 2005). Inspired by this, the ‘consciousness prior' in machine learning emphasizes a neural network architecture with a low-dimensional representation that arises from attention applied to an underlying high-dimensional state representation (Bengio, 2017). This low-D representation should efficiently represent the world at an abstract level such that it can be used to summarize and make predictions about future states. The presence of this attention-mediated bottleneck has a trickle-down effect that encourages disentangled representations at all levels such that they can be flexibly combined to guide actions and make predictions.

Conscious attention is required for the learning of many complex skills such as playing a musical instrument. However once fully learned, these processes can become automatic, possibly freeing attention up to focus on other things (Treisman et al., 1992). The mechanisms of this transformation are not entirely clear but insofar as they seem to rely on moving the burden of the task to different, possibly lower/more reflexive brain areas, it may benefit artificial systems to have multiple redundant pathways that can be engaged differently by attention (Poldrack et al., 2005).

### 4.4. Limitations of Attention: Bugs or Features?

Biological attention does not work perfectly. As mentioned above, performance can suffer when switching between different kinds of attention, arousal levels need be just right in order to reach peak performance, and top-down attention can be interrupted by irrelevant but salient stimuli. A question when transferring attention to artificial systems is are these limitations bugs to be avoided or features to be incorporated?

Distractability, in general, seems like a feature of attention rather than a bug. Even when attempting to focus on a task it is beneficial to still be aware of—and distractable by—potentially life-threatening changes in the environment. The problem comes only when an agent is overly distractable to inputs that do not pose a threat or provide relevant information. Thus, artificial systems should balance the strength of top down attention such that it still allows for the processing of unexpected but informative stimuli. For example, attentional blink refers to the phenomenon wherein a subject misses a second target in a stream of targets and distractors if it occurs quickly after a first target (Shapiro et al., 1997). While this makes performance worse, it may be necessary to give the brain time to process and act on the first target. In this way, it prevents distractability to ensure follow through.

Any agent, artificial or biological, will have some limitations on its energy resources. Therefore, prudent decisions about when to engage in the world versus enter an energy-saving state such as sleep will always be of relevance. For many animals sleep occurs according to a schedule but, as was discussed, it can also be delayed or interrupted by attention-demanding situations. The decision about when to enter a sleep state must thus be made based on a cost-benefit analysis of what can be gained by staying awake. Because sleep is also known to consolidate memories and perform other vital tasks beyond just energy conservation, this decision may be a complex one. Artificial systems will need to have an integrative understanding of their current state and future demands to make this decision.

## 5. Conclusions

Attention is a large and complex topic that sprawls across psychology, neuroscience, and artificial intelligence. While many of the topics studied under this name are non-overlapping in their mechanisms, they do share a core theme of the flexible control of limited resources. General findings about flexibility and wise uses of resources can help guide the development of AI, as can specific findings about the best means of deploying attention to specific sensory modalities or tasks.

## Author Contributions

GL conceived and wrote the article and generated the figures.

### Conflict of Interest

The author declares that the research was conducted in the absence of any commercial or financial relationships that could be construed as a potential conflict of interest. The reviewer MR declared a past co-authorship with the author GL to the handling Editor.
