# Corrigendum: Attention in Psychology, Neuroscience, and Machine Learning

**DOI:** 10.3389/fncom.2021.698574

**Published:** 2021-05-26

**Authors:** Grace W. Lindsay

**Affiliations:** Gatsby Computational Neuroscience Unit, Sainsbury Wellcome Centre, University College London, London, United Kingdom

**Keywords:** attention, artificial neural networks, machine learning, vision, memory, awareness

In the original article, there was an error. A portion of the text was repeated unnecessarily.

A correction has been made to *4. Ideas for Future Interaction Between Artificial and Biological Attention, 4.2. How to Deploy Attention, Paragraph 4*. The corrected paragraph is shown below.

Activities would likely need to flexibly decide which of several possible goals should be achieved at any time and therefore where attention should be placed. This problem clearly interacts closely with issues around reinforcement learning—particularly hierarchical reinforcement learning which involves the choosing of subtasks—as such decisions must be based on expected positive or negative outcomes. Indeed, there is a close relationship between attention and reward as previously rewarded stimuli attract attention even in contexts where they no longer provide reward (Camara et al., 2013). A better understanding of how humans choose which tasks to engage in and when should allow human behavior to inform the design of a multi-task AI.

The author apologizes for this error and states that this does not change the scientific conclusions of the article in any way. The original article has been updated.
